# Engineering the hinge region of human IgG1 Fc-fused bispecific antibodies to improve fragmentation resistance

**DOI:** 10.1038/s41598-018-35489-y

**Published:** 2018-11-22

**Authors:** Saori Suzuki, Hiroaki Annaka, Shota Konno, Izumi Kumagai, Ryutaro Asano

**Affiliations:** 1 Department of Biotechnology and Life Science, Graduate School of Engineering, Tokyo University of Agriculture and Technology, Tokyo, 184-8588 Japan; 2 CMIC JSR Biologics Co., Ltd., Shizuoka, 428-0013 Japan; 3 Department of Biomolecular Engineering, Graduate School of Engineering, Tohoku University, Sendai, 980-8579 Japan

## Abstract

Fc domain fusion can improve the therapeutic effects of relatively small biological molecules such as peptides, cytokines, and antibody fragments. Fc fusion proteins can also be used to enhance the cytotoxic effects of small bispecific antibodies (bsAbs). However, fragmentation of Fc fusion proteins, which mainly occurs around the hinge regions during production, storage, and circulation in the blood, is a major issue. In this study, we first investigated the mechanisms of fragmentation around the hinge region during storage using Fc-fused bsAbs with specificity for epidermal growth factor receptor and CD3 as a model. The fragmentation peaks generated by gel filtration analysis indicated that both contaminating proteases and dissolved active oxygen should be considered causes of fragmentation. We designed and constructed variants by introducing a point mutation into the upper hinge region, which reduced the cleavage caused by dissolved active oxygen, and shortened the hinge region to restrict access of proteases. These hinge modifications improved fragmentation resistance and did not affect the biological activity of the bsAbs *in vitro*. We confirmed the versatility of the hinge modifications using another Fc-fused bsAb. Our results show that hinge modifications to the Fc fusion protein, especially the introduction of a point mutation into the upper hinge region, can reduce fragmentation substantially, and these modifications can be used to improve the fragmentation resistance of other recombinant Fc fusion proteins.

## Introduction

Over the past 30 years, more than 60 antibody-based therapeutics and fusion proteins have been approved by the United States Food and Drug Administration (FDA) as promising agents for a variety of difficult-to-cure diseases^1^. Several antibody engineering techniques such as humanization, affinity maturation, and drug conjugation have made a remarkable contribution to their development. However, the construction of recombinant antibodies with high medical efficacy is desirable, especially in the field of cancer therapy. Consequently, many therapeutic strategies and non-natural antibody formats have been designed by domain reconstitution of antibody fragments and/or other functional protein domain^2,3^. One important approach is the construction of bispecific antibodies (bsAbs) that simultaneously bind to two different targets. For instance, bsAbs with specificity for cancer cells and various immune cells such as cytotoxic T cells can induce potent therapeutic effects by crosslinking these target cells. To date, only two fully non-natural antibody formats have been approved for clinical use, and both are bsAbs designed to recruit T cells against tumor cells^4^.

Fc fusion proteins have been constructed to improve the effectiveness of relatively small biological molecules such as cytokines and antibody fragments. It is expected that such biological molecules will have prolonged serum half-lives, because they have greater molecular weights and have binding capability to neonatal Fc receptors (FcRns)^5^. Fc fusion confers several other advantages: bivalency (because Fc generally forms stable homodimers); improved purification capability through protein A; and Fc-mediated effector functions such as antibody-dependent cell-mediated cytotoxicity (ADCC). Recombinant technology enables the generation of small bsAb fragments constructed from two different antibody-binding domains such as variable fragments (Fvs) and single-chain Fvs (scFvs). These bsAb fragments include diabodies (Dbs)^6^, single-chain diabodies (scDbs)^7^, tandem scFvs (taFvs)^8^, and minibodies (dimeric scDb-CH3 fusion proteins)^9^. Compared with classical bsAbs, small bsAb fragments have a convenient size for rapid tissue penetration and high target retention^10,11^; however, their rapid blood clearance and monovalency may limit their therapeutic applicability. Therefore, in the case of small bsAb fragments, the construction of Fc fusion proteins can help overcome these negative aspects^12,13^.

The fragmentation of intact antibodies and Fc fusion proteins during production, storage, and circulation in the blood is a major problem^14,15^. Consequently, several analytical methods for the detection of fragmentation, which mainly occurs around the hinge region, have been described. Such methods include: sodium dodecyl sulfate polyacrylamide gel electrophoresis (SDS-PAGE), size exclusion chromatography (SEC), capillary electrophoresis with SDS (CE-SDS)^16^, etc. In addition, strategies to prevent fragmentation have been also reported, e.g. regulation of temperature, pH, harvest time, and cell viability during cultivation, and consideration of addition protease inhibiters throughout the entire production process^17,18^. In addition to proteolytic fragmentation, dissolved active oxygen causes fragmentation around the hinge region of human IgG1^19^. This fragmentation can be reduced by introducing a point mutation into the upper hinge region of the IgG1^20^.

**Figure 1:**
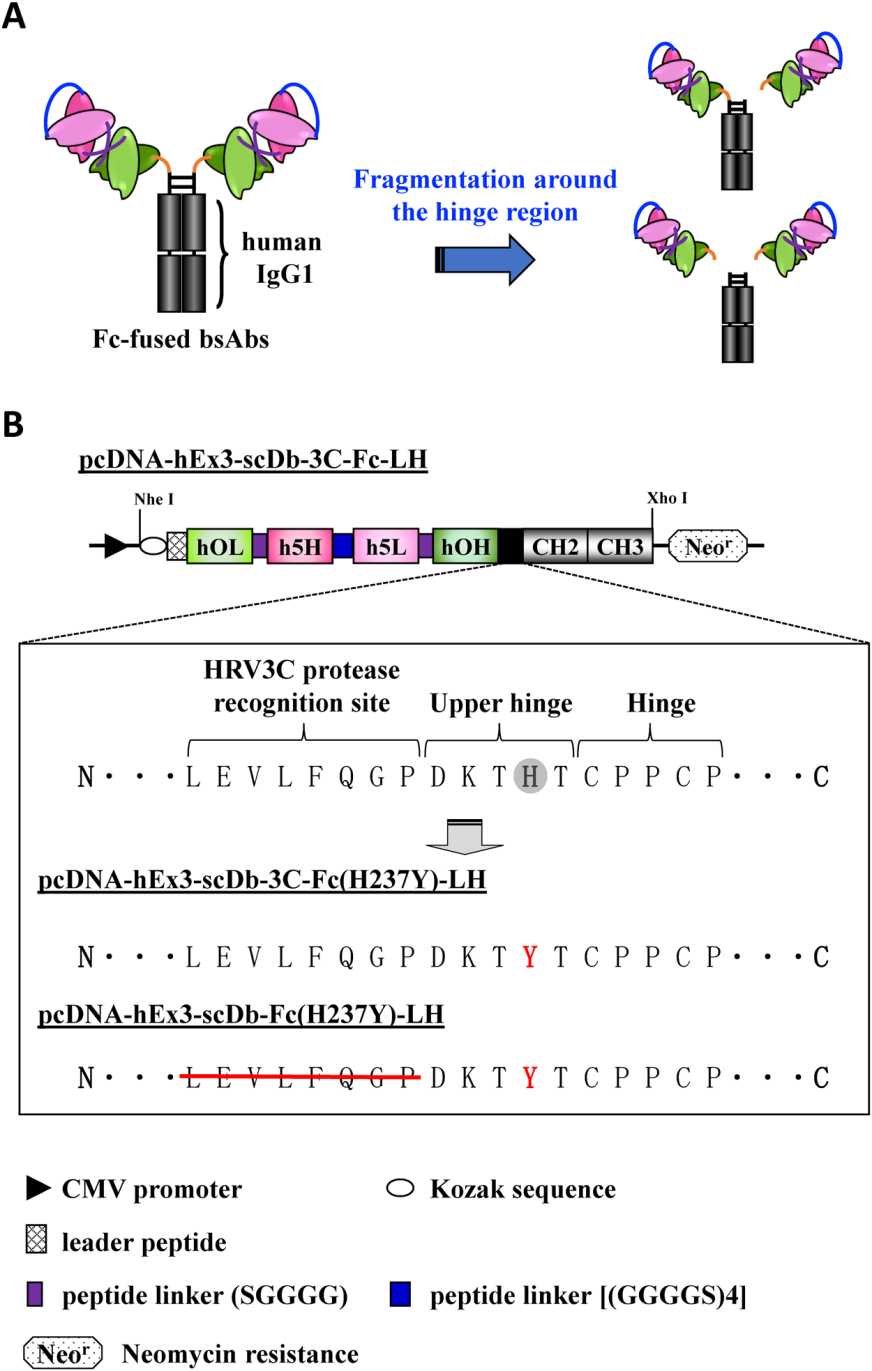
hEx3-scDb-3C-Fc-LH hinge designed to reduce fragmentation. (**A**) Schematic illustration of the fragmentation around the hinge region of Fc-fused bispecific antibodies (bsAbs) during storage. (**B**) Schematic diagrams of the expression vectors for hEx3-scDb-3C-Fc-LH, hEx3-scDb-3C-Fc(H237Y)-LH, and hEx3-scDb-Fc(H237Y)-LH. h5H and h5L, the VH and VL regions of the humanized anti-epidermal growth factor receptor (EGFR) antibody 528; hOH and hOL, the VH and VL regions of the humanized anti-CD3 antibody OKT3. The mutation site is written in red.

We have also focused on the development of bispecific antibodies for cancer treatment^21,22^ and have reported the marked *in vitro* and *in vivo* anti-tumor activity of hEx3-Db, a humanized bispecific Db targeting epidermal growth factor receptor (EGFR) and CD3^23^. Construction of the Fc fusion proteins also resulted in the cytotoxic enhancement of hEx3-Db^24^. We have also recently discovered that the domain rearrangement of bispecific Db led to substantial cytotoxic enhancement^25,26^, and the Fc fusion format based on the domain-rearranged variant of hEx3-Db, designated hEx3-scDb-3C-Fc-LH, also had higher *in vitro* and *in vivo* anti-tumor activity than that of the previous version^27^. Interestingly, this rearrangement also improved the fragmentation resistance and pharmacokinetics; however, there was still gradual fragmentation around the hinge region of hEx3-scDb-3C-Fc-LH during long-term storage (Fig. 1A).

In the present study, we first investigated the mechanisms causing the fragmentation of hEx3-scDb-3C-Fc-LH during storage. We then designed and constructed variants by introducing a point mutation into the upper hinge region to reduce the cleavage caused by dissolved active oxygen^20^ and shortening the hinge region to reduce non-specific digestion by proteases. These hinge modifications improved fragmentation resistance without affecting the biological activity of the antibody. We also confirmed the versatility of the modifications using the original Fc fusion format of hEx3-Db, i.e., hEx3-scDb-3C-Fc-HL. The results showed that modification of the hinges of Fc fusion proteins, especially introduction of a point mutation into the upper hinge region, can substantially reduce fragmentation. These modifications may improve fragmentation resistance in other recombinant Fc fusion proteins.

## Results

### Mechanisms that cause fragmentation around the hinge region of hEx3-scDb-3C-Fc-LH

**Figure 2:**
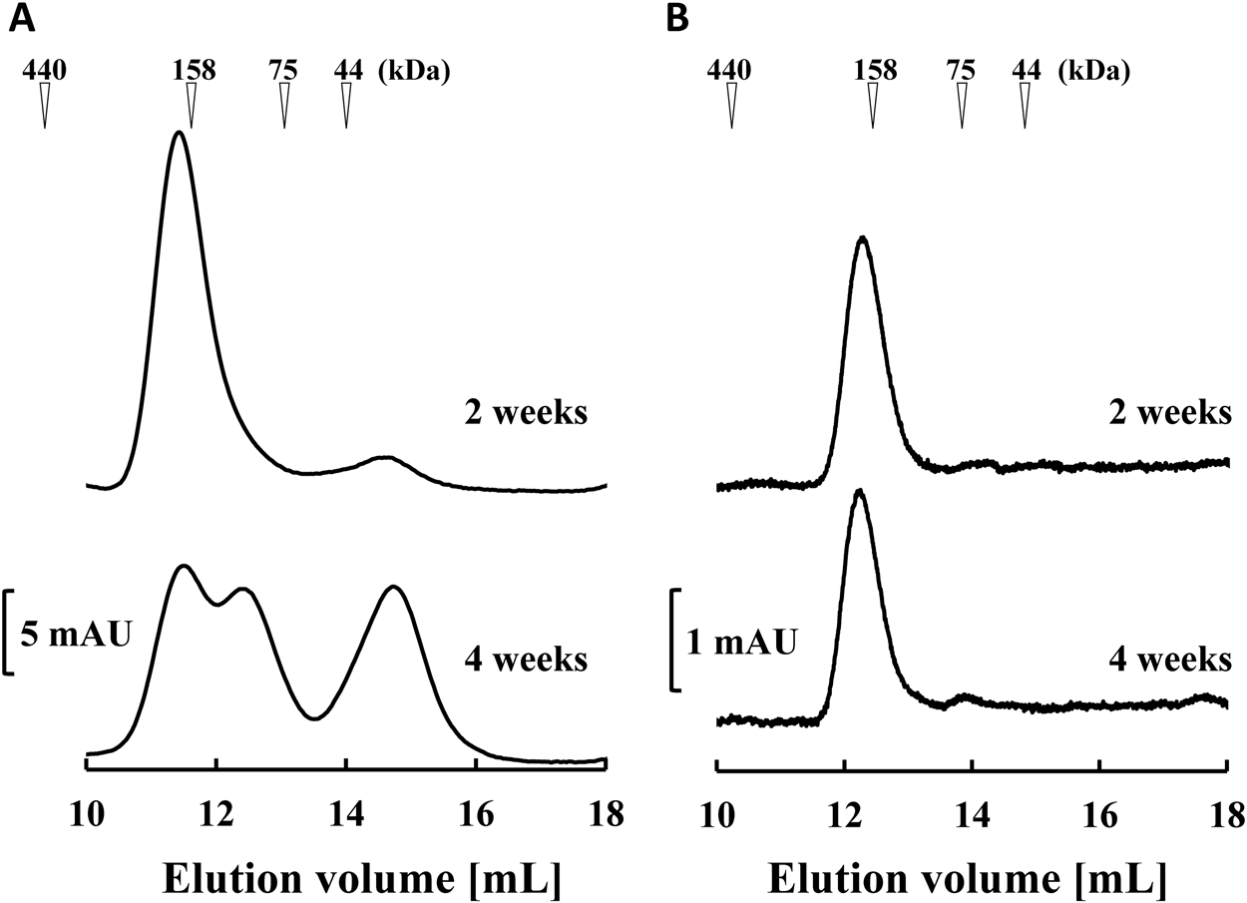
Gel filtration of hEx3-scDb-3C-Fc-LH to assess its storage stability. Fractionated hEx3-scDb-3C-Fc-LHs were stored under non-sterile conditions at 4 °C (**A**) or under sterilized conditions at 25 °C (**B**), then applied to a Superdex 200 10/300 GL column after 2- and 4-week storage. The calculated molecular mass is 158 kDa.

**Figure 3:**
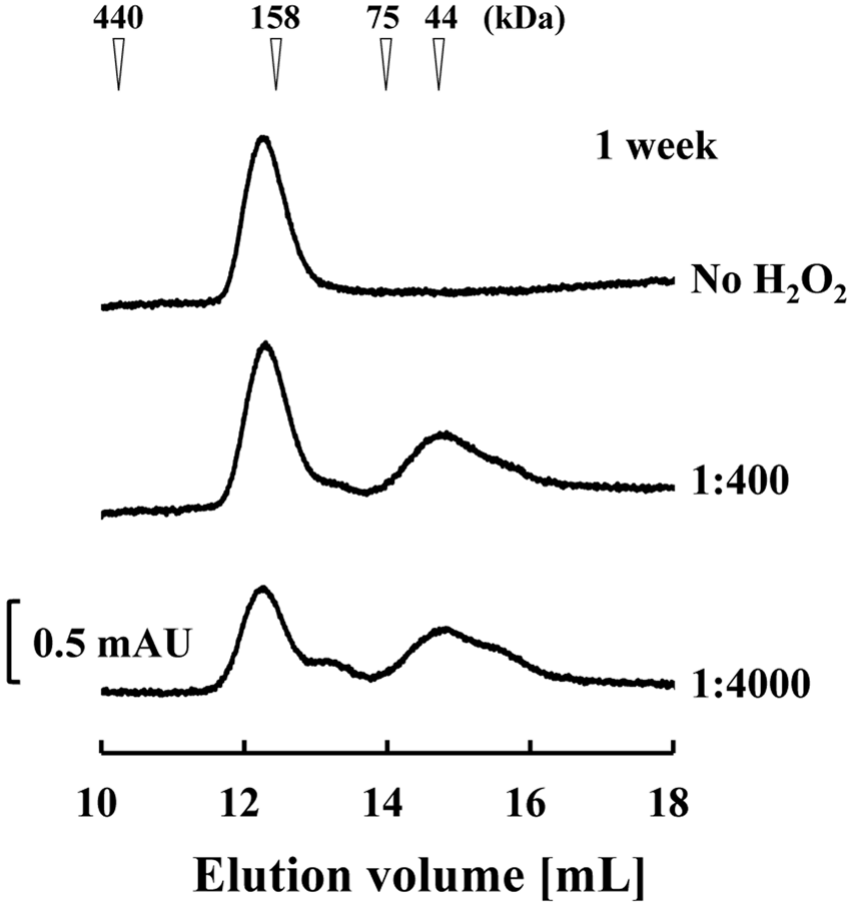
Gel filtration of hEx3-scDb-3C-Fc-LH to assess H_2_O_2_-mediated radical hinge fragmentation. Fractionated hEx3-scDb-3C-Fc-LHs were stored for 1 week under sterile conditions with H_2_O_2_ at 25 °C, then applied to a Superdex 200 10/300 GL column. The molar ratios of the antibody to H_2_O_2_ were 1:400 or 1:4,000.

We evaluated the molecular structures of hEx3-scDb-3C-Fc-LH by gel filtration analysis using fractionated monomers to determine the mechanisms that caused fragmentation around the hinge region of hEx3-scDb-3C-Fc-LH during storage. The HRV3C protease recognition site in hEx3-scDb-3C-Fc-LH can also be susceptible to non-specific digestion by proteases from contaminating bacteria or expression host cell, and this type of proteolytic digestion is usually temperature-dependent. Therefore, we performed the analyses under two storage conditions: non-sterilized condition at 4 °C and sterilized condition at 25 °C. Single peaks corresponding to the monomer were observed under both conditions after storage for 2 weeks; however, several peaks were attributable to fragmented species which emerged mainly in the non-sterilized group after storage for 4 weeks (Fig. 2A,B). In addition to proteolytic fragmentation, dissolved active oxygen can cause fragmentation around the hinge region of human IgG1, and this can be promoted by adding H_2_O_2_^19^. Even under sterile conditions, obvious fragmentation peaks were observed in the presence of H_2_O_2_ after storage for only 1 week (Fig. 3). These results show that both contaminating proteases and dissolved active oxygen should be considered causes of fragmentation around the hinge region of hEx3-scDb-3C-Fc-LH, as in human IgG1.

### Design of the hinge region of hEx3-scDb-3C-Fc-LH for fragmentation resistance

**Figure 4:**
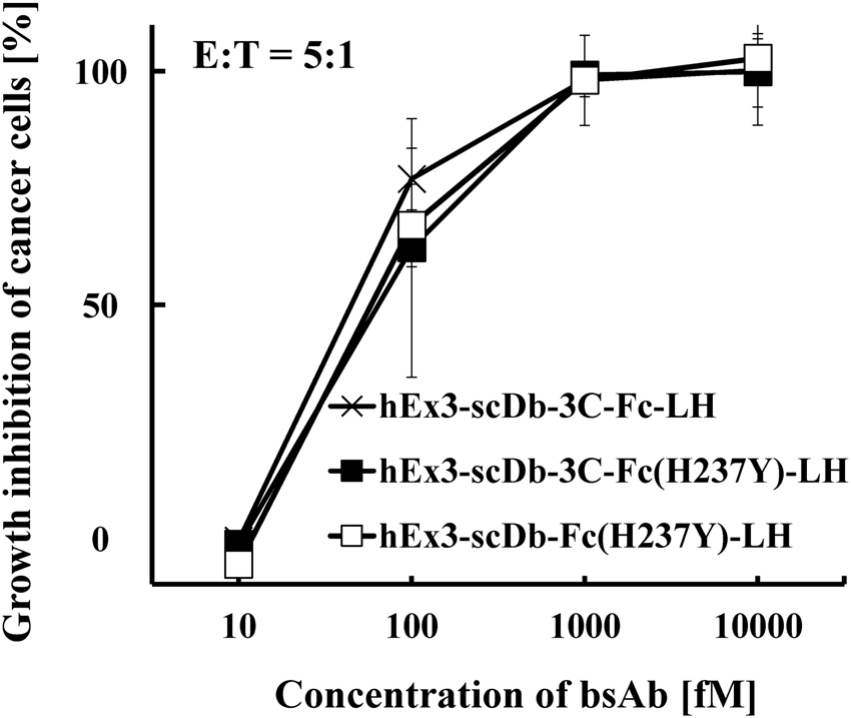
Growth inhibition of epidermal growth factor receptor (EGFR)-positive TFK-1 cells by bispecific antibodies (bsAbs). Lymphokine-activated killer cells with the T-cell phenotype (T-LAK) cells were added to TFK-1 cells at a ratio of 5:1 (n = 4). Data are presented as mean ± 1 standard deviation (s.d.).

To reduce the fragmentation of hEx3-scDb-3C-Fc-LH, we designed and prepared two mutants with modified hinges, as described in Methods (Fig. 1B). Briefly, we constructed hEx3-scDb-3C-Fc(H237Y)-LH by replacing the histidine residue (H) in the upper hinge region with tyrosine (Y) to reduce the fragmentation caused by dissolved active oxygen, according to the method described in the literature^20^. We also constructed hEx3-scDb-Fc(H237Y)-LH by eliminating the HRV3C protease recognition site in hEx3-scDb-3C-Fc(H237Y)-LH to reduce non-specific digestion by proteases. To evaluate the effects of the modifications of the hinge region of hEx3-scDb-3C-Fc-LH on the inhibition of human carcinoma cell growth, we analyzed each of the fractionated monomers by MTS tetrazolium assay. The hEx3-scDb-3C-Fc-LH mutants exhibited almost identical growth inhibition (Fig. 4), indicating that the modifications of the hinge region had little or no effect on the biological activity of hEx3-scDb-3C-Fc-LH *in vitro*.

### Effect of the hEx3-scDb-3C-Fc-LH hinge modifications on fragmentation

**Figure 5:**
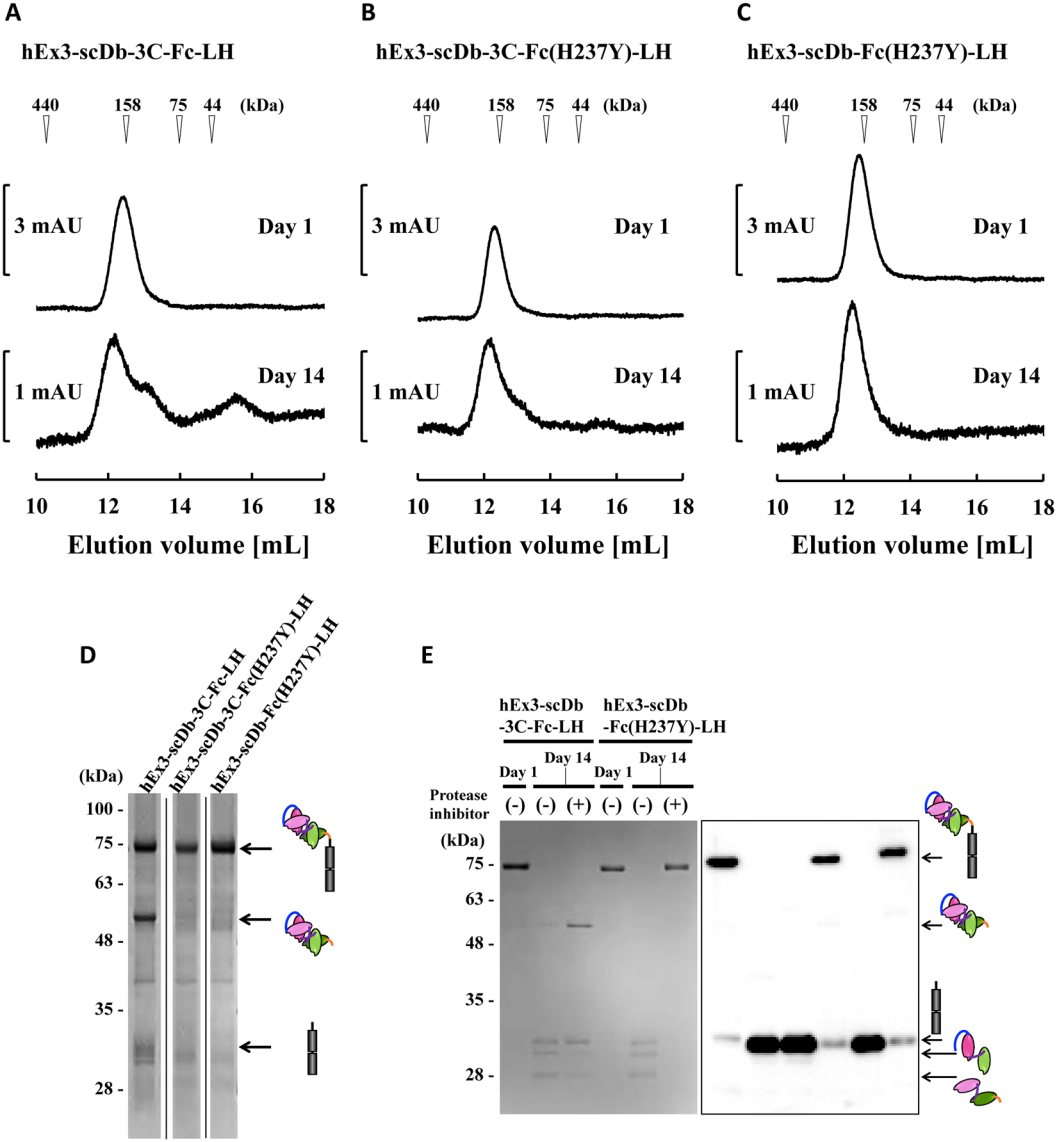
Gel filtration of bispecific antibodies (bsAbs) to assess hinge fragmentation mediated by the addition of H_2_O_2_. The fractionated bsAbs were stored for 2 weeks under sterile conditions at 25 °C in the presence of 60 mM H_2_O_2_, then applied to a Superdex 200 10/300 GL column (**A**–**C**). (**D**) Reducing sodium dodecyl sulfate-polyacrylamide gel electrophoresis (SDS-PAGE) applied to each bsAb after storage for 17 days. The calculated molecular masses for single-chain diabodies (scDb) and Fc region generated from fragmentation are 53 kDa and 26 kDa, respectively. Notes that the band for Fc region was shifted to around 30 kDa by glycosylation. The full-sized image is presented in Supplementary Fig. 2. (**E**) Western blotting using horseradish peroxidase (HRP)-conjugated anti-human IgG Fc. Fractionated hEx3-scDb-3C-Fc-LH and hEx3-scDb-Fc(H237Y)-LH were stored for 2 weeks under non-sterile condition at 25 °C with or without a protease inhibitor cocktail (Nacalai Tesque, Inc. Kyoto, Japan), then applied to reducing SDS-PAGE.

To confirm the influence of the hinge modifications on the fragmentation of hEx3-scDb-3C-Fc-LH, we incubated each of the sterilized fractionated monomers in the presence of H_2_O_2_ at 25 °C and investigated the molecular structures using gel filtration chromatography and SDS-PAGE. Both mutants exhibited fragmentation resistance under such conditions. There was substantial fragmentation in hEx3-scDb-3C-Fc-LH and marginally greater resistance to fragmentation in hEx3-scDb-Fc(H237Y)-LH (Fig. 5A–C). Fragmentation ratios of main peaks to low molecular weights calculated from peak areas separated by base lines supported these findings (Supplementary Fig. 1 and Table 1). SDS-PAGE under reducing conditions also revealed the fragmentation resistance of the mutants after storage for 17 days (Fig. 5D). The bands observed at approximately 50 kDa and 30 kDa in hEx3-scDb-3C-Fc-LH were attributable to the scDb and Fc regions, respectively, indicating that fragmentation occurred mainly around the hinge region, as expected. Then, to investigate effects of these two modifications in more detail, we performed Western blot analysis (Fig. 5E). Fractionated hEx3-scDb-3C-Fc-LH and hEx3-scDb-Fc(H237Y)-LH were stored for 2 weeks under non-sterile condition at 25 °C with or without of a protease inhibitor cocktail. Severe fragmentation including digestion of a middle linker in the scDb portion was observed in both bsAbs in the absence of the protease inhibitor. Addition of the protease inhibitor reduced the fragmentation of the middle linker for hEx3-scDb-3C-Fc-LH, but it was not effective for the fragmentation around the hinge region. In contrast, almost complete suppression for the fragmentation of hEx3-scDb-Fc(H237Y)-LH was observed by the addition of protease inhibitor. These results show that hinge modifications, especially H237Y mutations are important to reduce fragmentation and may increase fragmentation resistance in other recombinant Fc fusion proteins.

### Application of the hinge modification to hEx3-scDb-3C-Fc-HL

**Figure 6:**
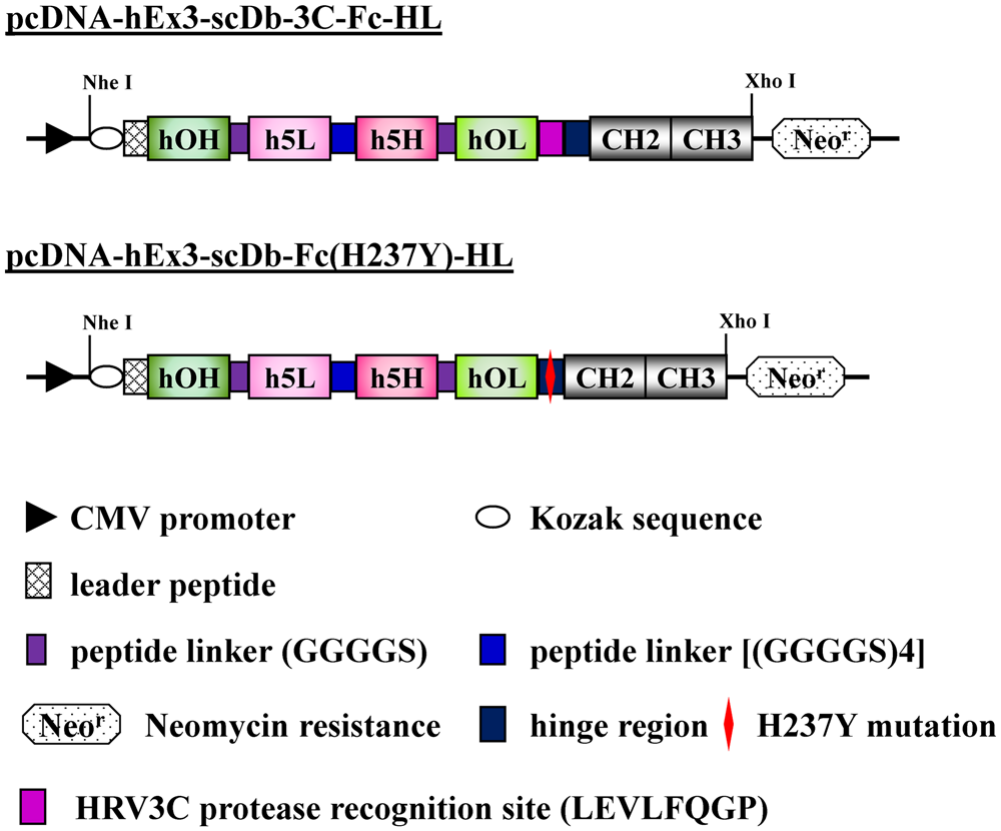
Schematic diagrams of the expression vectors for hEx3-scDb-3C-Fc-HL and hEx3-scDb-Fc(H237Y)-HL.

**Figure 7:**
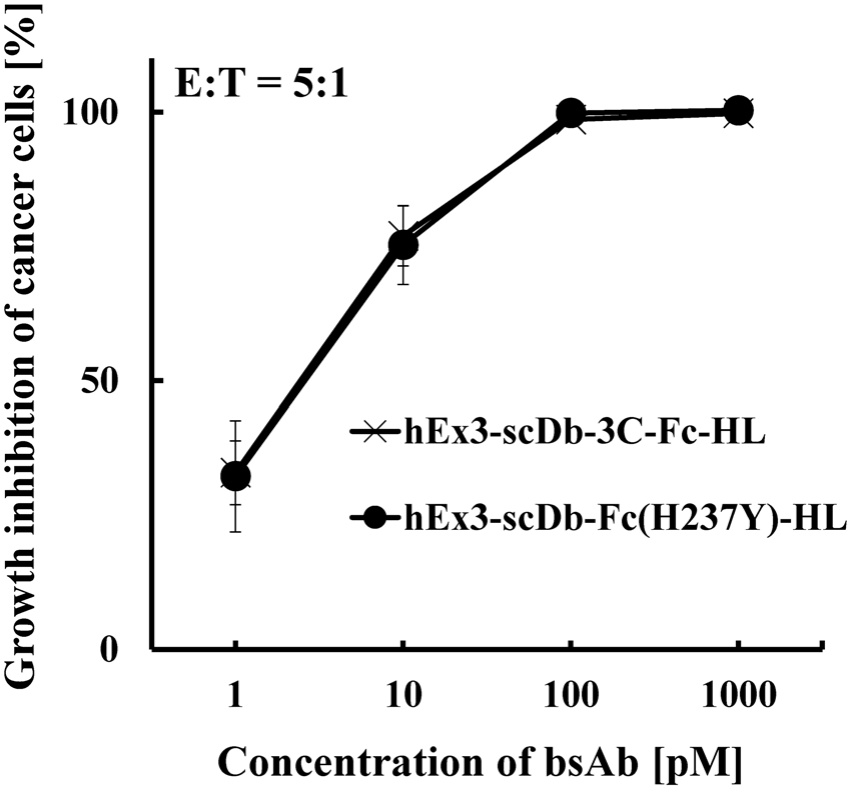
Growth inhibition of epidermal growth factor receptor (EGFR)-positive TFK-1 cells by bsAbs. Lymphokine-activated killer cells with the T-cell phenotype (T-LAK) cells were added to TFK-1 cells at a ratio of 5:1. (n = 4). Data are presented as mean ± 1 standard deviation (s.d.) and are representative of at least three independent experiments.

**Figure 8:**
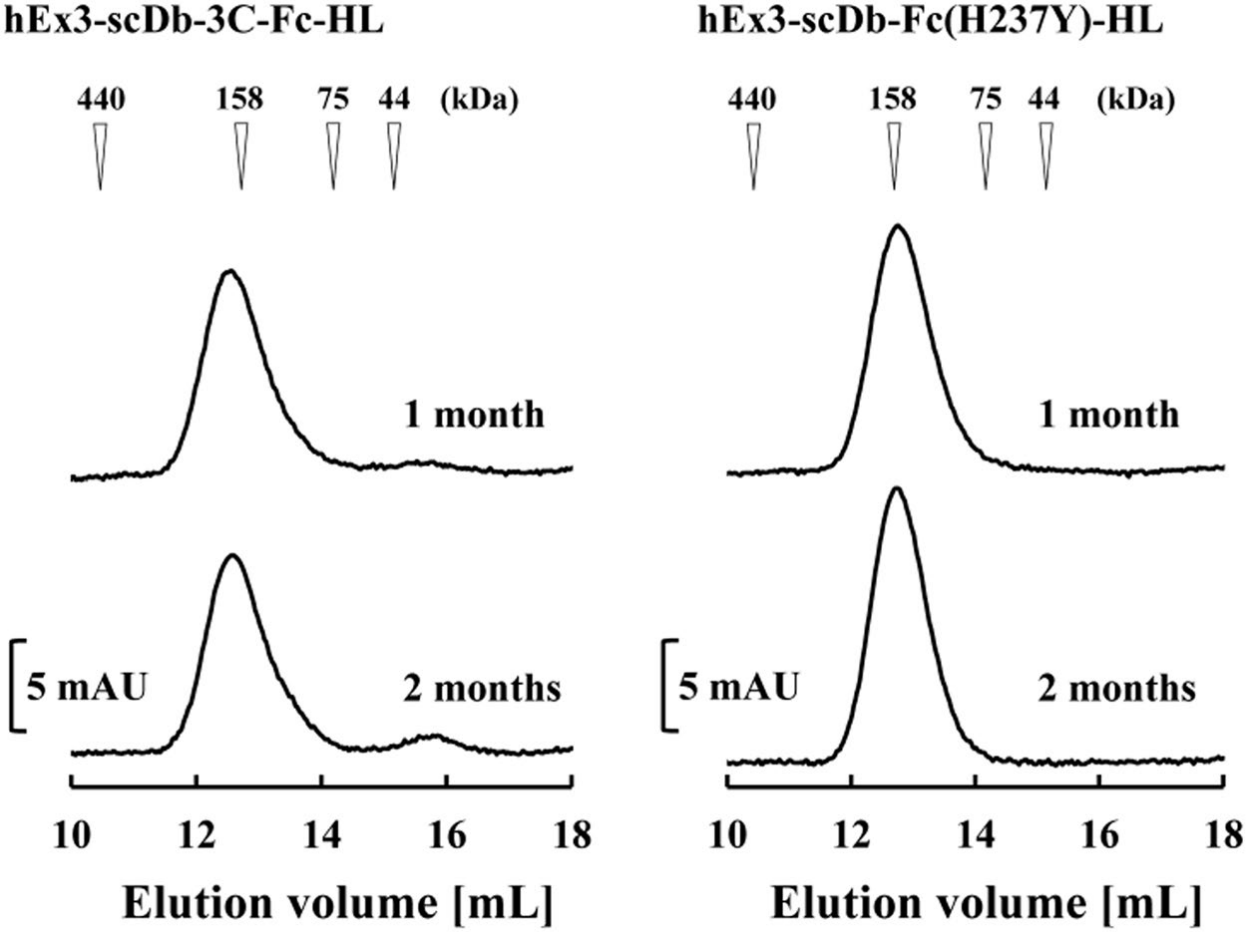
Gel filtration of the bispecific antibodies (bsAbs) to assess their stability after storage. The fractionated bsAbs were stored for 1 and 2 months under sterile conditions at 4 °C, then applied to a Superdex 200 10/300 GL column.

We previously reported hEx3-scDb-3C-Fc-HL, which had a different domain order than the LH type and exhibited lower fragmentation resistance than the LH type^27^. To investigate the versatility of the hinge modifications for other Fc fusion proteins, we constructed hEx3-scDb-Fc(H237Y)-HL by introducing a His/Tyr mutation and eliminating the HRV3C protease recognition site, similar to the construction of hEx3-scDb-Fc(H237Y)-LH (Fig. 6). To evaluate the effects of the hinge modifications of hEx3-scDb-3C-Fc-HL on the inhibition of human carcinoma cell growth, we investigated the fractionated monomers using MTS. In the case of hEx3-scDb-3C-Fc-HL, these modifications also had no effect on the biological activity *in vitro* (Fig. 7). Further, comparable kinetic parameters were observed in both bsAbs by surface plasmon resonance spectroscopy (Supplementary Fig. 3 and Table 2). To evaluate the influence of the hinge modifications on the fragmentation of hEx3-scDb-Fc(H237Y)-HL during long-term storage, we incubated the sterilized fractionated monomers without H_2_O_2_ at 25 °C and investigated them using gel filtration chromatography. Fragmentation was almost completely eliminated in hEx3-scDb-Fc(H237Y)-HL compared with hEx3-scDb-3C-Fc-HL, as with hEx3-scDb-Fc(H237Y)-LH (Fig. 8). These results demonstrate that, in addition to eliminating the risk from contaminating proteases, hinge modifications are a practical way of increasing fragmentation resistance, and these modifications, specifically the H237Y mutation, may be used to improve other recombinant Fc fusion proteins.

## Discussion

Advances in recombinant technology have made it feasible to fuse Fc proteins to receptors^28^, ligands^29^, enzymes^30^, and peptides^31^ to improve their therapeutic effects. Enbrel® (etanercept) was approved by the FDA in 1998^32^ and was the first Fc fusion biological molecule to receive such recognition. It is constructed by linking an extracellular ligand-binding portion of the human tumor necrosis factor receptor (TNFR) to human IgG1 Fc and is a typical example of an Fc fusion protein. Fc fusion greatly improved the therapeutic effects of the TNFR monomer by increasing its binding affinity to TNF and prolonging its serum half-life^33^. The functionalization of small bsAbs by the construction of Fc fusion formats has also been reported^3^. We found that hEx3-Fc was approximately ten times more effective at inhibiting cancer growth than hEx3-Db^24,34^.

During the construction of Fc fusion proteins, the hinge region of the antibody is generally grafted together to ensure high flexibility while retaining the function of the antibody and its accessibility to the target^35^. Furthermore, disulfide bonds in the hinge region are necessary for the formation of stable dimers, and it has also been reported that the hinge region contributed to ADCC activity^36^. However, the flexibility of the hinge region also facilitates access to contaminating proteases, resulting in fragmentation during production^37,38^. Additionally, various non-enzymatic IgG fragmentation mechanisms have also been proposed, e.g., β-elimination^39^, direct hydrolysis^40^, copper-mediated cleavage^41^, and free-radical catalysis^19^. Therefore, to reduce such fragmentation, various factors, including the running conditions, the storage buffer, and the additives used during production have been widely investigated^42^.

In the present study, we first investigated the mechanisms that caused fragmentation around the hinge region of hEx3-scDb-3C-Fc-LH, the Fc fusion format of hEx3-Db. Marked fragmentation was observed under non-sterile conditions, indicating that hEx3-scDb-3C-Fc-LH was fragmented proteolytically (Fig. 2A,B). In contrast, the addition of H_2_O_2_ promoted fragmentation, even under sterile conditions (Fig. 3), indicating that the hEx3-scDb-3C-Fc-LH was also fragmented by free-radicals from dissolved active oxygen, as in human IgG1 with the same amino acid sequence in the hinge region^19^. We then introduced a H237Y mutation into the upper region of the hinge to reduce the cleavage caused by dissolved active oxygen and shortened the hinge region to hamper protease access and/or reduce potential cleavage site. It has been reported that longer connection linkers between protein domains were more likely to be exposed to the solvent^43^. These hinge modifications did not affect the inhibition of cancer growth (Fig. 4), and the H237Y mutation substantially improved fragmentation resistance in the presence of H_2_O_2_, as expected (Fig. 5). In contrast, shortening the hinge region showed only the marginal effects on fragmentation resistance. The hinge region is involved in Fc-mediated effector functions^36,44,45^. In fact, a H237Y mutation for fragmentation resistance in the upper hinge region also enhances ADCC activity in human IgG1^20^. Although in the case of hEx3-scDb-3C-Fc-LH we have reported that Fc-mediated effector functions were not necessary for effective anti-tumor activity^27^, in many other cases enhancement of those functions is expected to lead to improvements in therapeutic effects.

We also successfully confirmed the versatility of the hinge modifications using another Fc fusion format of hEx3-Db, i.e., hEx3-scDb-3C-Fc-HL (Figs 7 and 8). We have previously reported that hEx3-scDb-3C-Fc-HL has lower fragmentation resistance than that of the LH type^27^. Although the reason for this difference remains unknown, it may be due to the rearrangement of the domain order, which alters the surface that is accessible to proteases. Furthermore, hEx3-scDb-3C-Fc-LH exhibited a prolonged *in vivo* half-life compared with the HL type^27^. Mabry *et al*. (2010) have also reported that differences in configuration affected the *in vivo* half-life of Fc-fused bsAbs, even though each comprised identical antibody elements similar to those in the present study^46^. Our results suggest that proteolytic fragmentation resistance may contribute to the differences in *in vivo* half-life. The interaction with FcRn is also involved in *in vivo* half-life^47^. Differences in the net charge of the fusion molecules and/or their steric hindrance with regard to Fc affect this interaction, and, consequently, the half-life. Although Fc fusion proteins have shorter *in vivo* half-lives than that of IgG^48^, we confirmed that hEx3-scDb-3C-Fc-LH had a comparable *in vivo* half-life to that of IgG molecules.

Brezski *et al*. (2010) have well reviewed the cleavage of IgGs by proteases^49^. They have listed the potential cleavage points around the hinge region, including lower hinge associated with FcγR binding, and mammalian and bacterial enzymes capable of cleaving human IgG1. In this study, we have focused on the proteolytic cleavage mediated by an enzyme with the recognition site around His237. However, actually fragmentation in the middle linker of the scDb portion was also observed (Fig. 5E). Thus, in accordance with previous reports, we are conducting a more detailed investigation of proteolytic fragmentation using examining protease digestion products and protease inhibitors. We are also attempting to determine the 3D structures of the hEx3-Db Fc fusion protein to obtain a better understanding of the importance of protease-accessible surfaces.

Contaminating proteases and dissolved active oxygen are possible causes of fragmentation around the hinge region of Fc-fused bsAbs, and hinge modifications, especially His/Tyr mutations, can substantially reduce fragmentation. Various Fc fusion proteins are being designed and developed, mainly for therapeutic purposes. The hinge modifications described in this paper may increase the stability of such molecules.

## Methods

### Construction of expression vectors for Fc-fused bsAbs with modified hinge regions

As in our previous report^23^, in the present study, we described the VH and VL regions of the humanized anti-EGFR antibody 528 as h5H and h5L, and those of the humanized anti-CD3 antibody OKT3 as hOH and hOL. We have previously described the construction of the mammalian expression vectors pcDNA-hEx3-scDb-3C-Fc-HL for hEx3-scDb-3C-Fc-HL^24^ and pcDNA-hEx3-scDb-3C-Fc-LH for hEx3-scDb-3C-Fc-LH^27^, in which a single-chain form of hEx3-Db was fused to a human IgG1 Fc region via a recognition site (LEVLFQGP) for human rhinovirus 3C (HRV3C) protease. Two chimeric single-chain components of hEx3-Db were connected via a 20-amino acid linker ((GGGGS)4) in the order hOHh5L-h5HhOL for HL and in the order hOLh5H-h5LhOH for LH. To prevent fragmentation, the H at 237 in the hinge region of hEx3-scDb-3C-Fc-LH was replaced by Y using overlap polymerase chain reaction (PCR), according to a method described in the literature^20^. The numbering of residues was based on the study of Kabat *et al*.^50^. Subsequently, pcDNA-hEx3-scDb-Fc(H237Y)-LH was constructed by eliminating the HRV3C recognition site of pcDNA-hEx3-scDb-3C-Fc(H237Y)-LH to reduce protease accessibility. The expression vector pcDNA-hEx3-scDb-Fc(H237Y)-HL for hEx3-scDb-Fc(H237Y)-HL was constructed in a similar way.

### Preparation of Fc-fused bsAbs

The methods used to express in Chinese hamster ovary (CHO) cells and purify Fc-fused bsAbs have been described previously^51^. Briefly, the Fc-fused bsAbs were first purified on a protein A column (GE Healthcare Bio-Science Corp., Piscataway, NJ, USA). Gel filtration analysis with a HiLoad Superdex 200 pg column (26/600; GE Healthcare) was used to fractionate the monomers of each bsAb. The column was equilibrated with phosphate-buffered saline (PBS), and protein A-purified bsAb was loaded onto the column at a flow rate of 2–2.5 mL/min. The long-term stability of bsAbs in storage was evaluated using a Superdex 200 GL column (10/300; GE Healthcare). The column was equilibrated with PBS, and purified bsAb was loaded onto the column at a flow rate of 0.5 mL/min. For Western blotting analysis, hEx3-scDb-Fc(H237Y)-LH was prepared using Expi293 Expression System (Thermo Fisher Scientific, Waltham, MA, USA).

### Confirmation of H_2_O_2_-mediated fragmentation of Fc-fused bsAbs

To accelerate fragmentation, 40 μM, 400 μM, or 60 mM H_2_O_2_ was added to 100 nM Fc-fused bsAbs in PBS at pH 7.5. After incubation at 25 °C, fragmentation was evaluated using a Superdex 200 GL column (10/300; GE Healthcare). The column was equilibrated with PBS, and 0.5 mL of the reaction mixture was loaded onto the column at a flow rate of 0.5 mL/min.

### Cell lines

Human bile duct carcinoma (TFK-1) was used in the present study. The TFK-1 cell line was established by our group^52^. The TFK-1 cell line was cultured in Roswell Park Memorial Institute 1640 (RPMI 1640) medium supplemented with 10% fetal bovine serum (FBS), 100 U/mL penicillin, and 100 μg/mL streptomycin.

### *In vitro* killing assay

Lymphokine-activated killer cells with the T-cell phenotype (T-LAK) were induced, as described in the literature^53^. Briefly, peripheral blood mononuclear cells (PBMCs) were cultured for 48 h at a density of 1 × 10^6^ cells/mL in medium supplemented with 100 IU/mL recombinant human IL-2 (Shionogi Pharmaceutical Co., Osaka, Japan) in a culture flask (A/S Nunc, Roskilde, Denmark) that was pre-coated with anti-CD3 monoclonal antibody (10 μg/mL). The *in vitro* growth inhibition of the cancer cells was assayed using a MTS assay kit (CellTiter 96® AQueous Non-Radioactive Cell Proliferation Assay; Promega), as reported in the literature^53^.

## Electronic supplementary material

Supplementary information
