# Supplementary material for: Engineering the hinge region of human IgG1 Fc-fused bispecific antibodies to improve fragmentation resistance
Source: Sci Rep. 2018 Nov 22;8:17253. doi: 10.1038/s41598-018-35489-y (PMC6250740; doi:10.1038/s41598-018-35489-y)
Supplement: Supplementary file 1 — Supplementary information [file 41598_2018_35489_MOESM1_ESM.pdf]

Supplementary information

Engineering the hinge region of human IgG1 Fc-fused bispecific antibodies to improve fragmentation resistance

Saori Suzuki[1], Hiroaki Annaka[2], Shota Konno[3], Izumi Kumagai[1,3], & Ryutaro Asano[1]*

[1]Department of Biotechnology and Life Science, Graduate School of Engineering, Tokyo University of Agriculture and Technology, Tokyo 184-8588, Japan

[2]CMIC JSR Biologics Co., Ltd., Shizuoka 428-0013, Japan

[3]Department of Biomolecular Engineering, Graduate School of Engineering, Tohoku University, Sendai 980-8579, Japan

Supplementary Figure 1

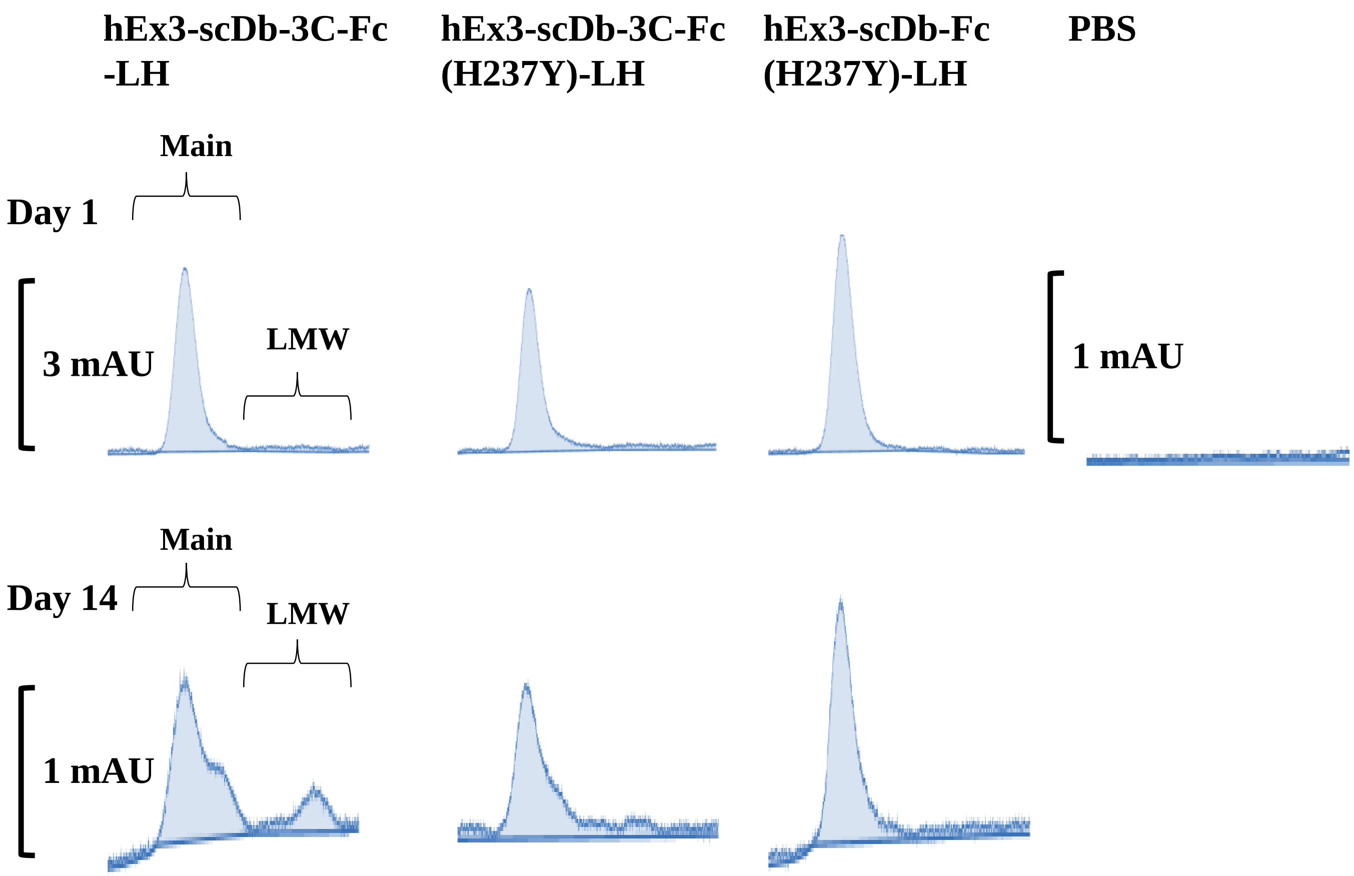

The law data of gel filtration for Fig. 5ABC with base line.

Supplementary Table 1

The fragmentation ratio of each bispecific antibody (bsAb) on Day 0 and 14. Note that our program could not distinguish shoulder peaks corresponded to partial degradation products from main peaks; therefore, in this table, main peaks contained the partial degradation products as indicated in Supplementary Figure 1.

	Ratio (LMW/Main)	
	**Day 1**	**Day 14**
**hEx3-scDb-3C-Fc-LH**	**0.067**	**0.24**
**hEx3-scDb-3C-Fc(H237Y)-LH**	**0.080**	**0.19**
**hEx3-scDb-Fc(H237Y)-LH**	**0.020**	**0.082**

LMW, low molecular weight
Main, peak corresponding to intact bsAb

Supplementary Figure 2

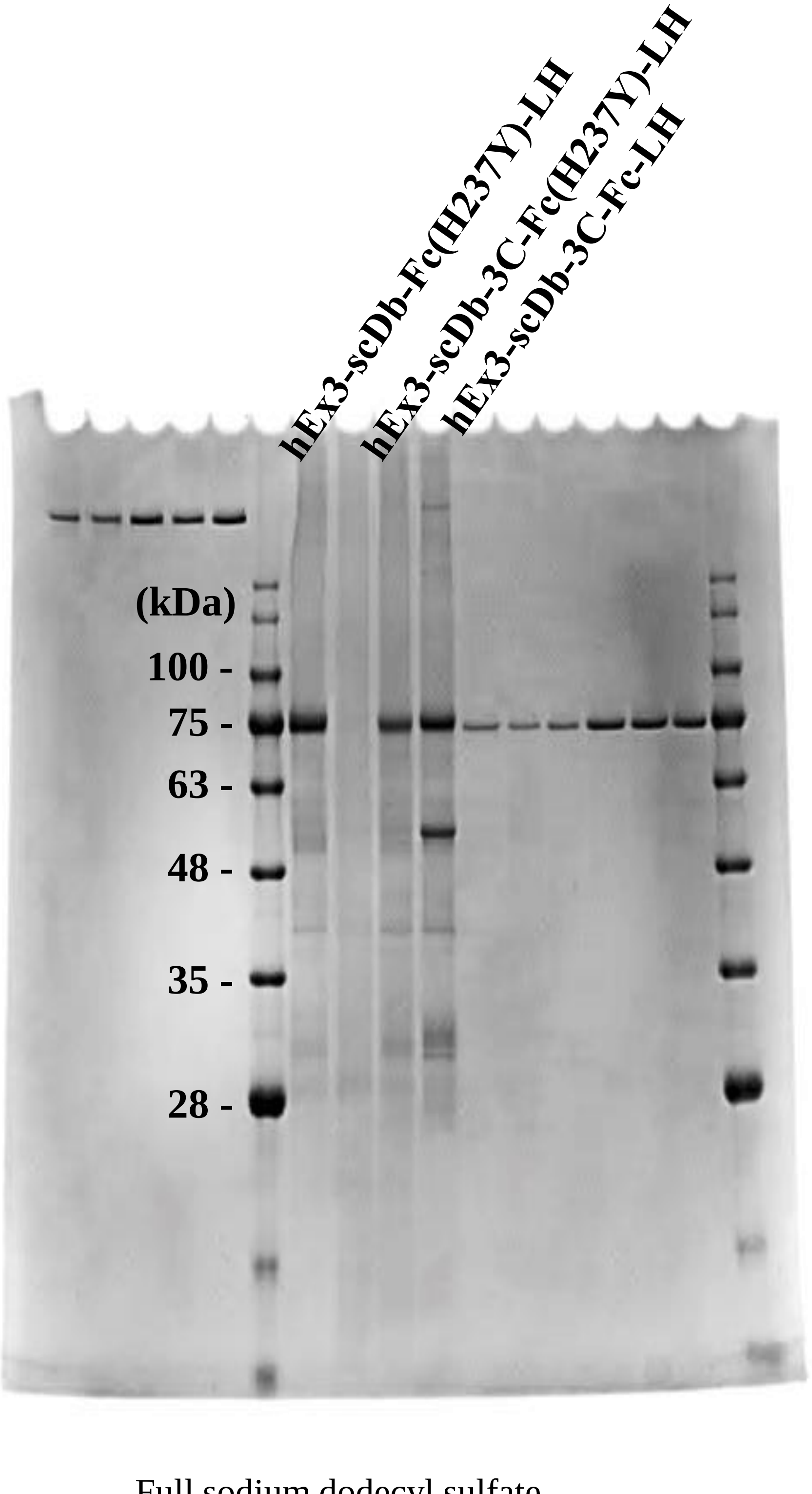

Full sodium dodecyl sulfate polyacrylamide gel electrophoresis (SDS-PAGE) image of Fig. 5D.

Supplementary Figure 3

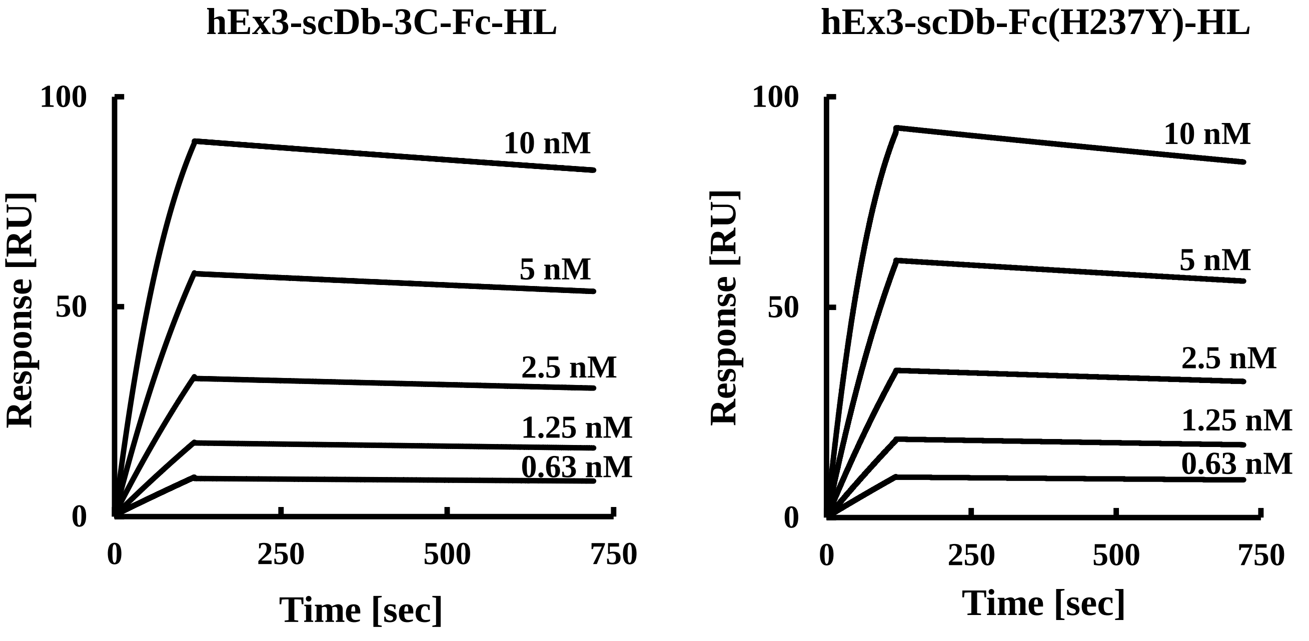

Surface plasmon resonance sensorgrams for bispecific antibodies (bsAbs). Soluble epidermal growth factor receptor (EGFR) was immobilised on a CM5 sensor chip, and various concentrations of bsAbs were then allowed to flow over the bound EGFR. The data were referenced by subtracting the response of a blocked blank cell.

Supplementary Table 2

Binding parameters. Kinetic parameters were calculated by means of a global fitting analysis with the assumption of a 1:1 Langmuir binding model.

	k_{on} (× 10^6 $M^{-1}s^{-1}$)	k_{off} (× 10^{-4} s^{-1})	K_D (× 10^{-10} M)
**hEx3-scDb-3C-Fc-HL**	**1.33**	**1.45**	**1.09**
**hEx3-scDb-Fc(H237Y)-HL**	**1.62**	**1.69**	**1.04**
